# Effect of different rice transplanting patterns on microbial community in water, sediment, and *Procambarus clarkii* intestine in rice-crayfish system

**DOI:** 10.3389/fmicb.2023.1233815

**Published:** 2023-08-10

**Authors:** Jin Huang, Jinghao Li, Wenzong Zhou, Yongxu Cheng, Jiayao Li

**Affiliations:** ^1^Key Laboratory of Integrated Rice-Fish Farming Ecosystem, Ministry of Agriculture and Rural Affairs, Shanghai Ocean University, Shanghai, China; ^2^Centre for Research on Environmental Ecology and Fish Nutrition (CREEFN) of the Ministry of Agriculture, Shanghai Ocean University, Shanghai, China; ^3^National Demonstration Center for Experimental Fisheries Science Education, Shanghai Ocean University, Shanghai, China; ^4^Eco-environmental Protection Research Institute, Shanghai Academy of Agricultural Sciences, Shanghai, China

**Keywords:** rice-crayfish farming, *Procambarus clarkii*, transplanting patterns, intestinal microbiota, microbial community

## Abstract

Although the microbial ecology of integrated rice-crayfish farming systems is receiving increasing attention with the expanding application area in China, the effects of rice transplanting patterns on the microbial community of water, sediment and *Procambarus clarkii* intestine in rice-crayfish system has yet to be determined. This study explored the microbial community present in water, sediment and intestine samples from three transplant patterns (rice crayfish with wide-narrow row transplanting, rice-crayfish with normal transplanting and pond-crayfish, abbreviated as RC-W, RC, and PC, respectively) using high-throughput sequencing. The results showed that the dominant microbial taxa from sediment, surrounding water, and intestine at phylum level were Proteobacteria, Chloroflexi, Cyanobacteria, Actinobacteria, Bacteroidetes. The patterns of rice transplanting had significant effects on microbial biodiversity and species composition in surrounding water. The OTUs community richness of water under RC group was significantly higher than that of PC group and RC-W group. The OTU relative abundance of top 10 operational taxonomic units had significantly different (*p* < 0.05) in the water samples from the three groups. The intestinal OTU community richness of *Procambarus clarkii* in the three groups was positively correlated with the community richness of water. The proximity between intestinal and water samples in PCA diagram indicated that their species composition was more similar. The results also showed that rice transplanting patterns can affect intestinal microbial biodiversity of *Procambarus clarkii* and the intestinal microbial biodiversity correlated with water bodies. Although the intestinal microbial diversity of crayfish in RC-W group was lower than that in RC group, the relative abundance of potential pathogenic bacteria, such as *Vibrio, Aeromonas*, in intestine of the crayfish in the RC-W group was significantly decreased under rice wide-narrow row transplanting model. Redundancy analysis revealed that environmental parameters, such as pH, DO, nitrate, which regulate the composition of microbial community structures. This study provides an understanding for microbial response to different rice transplanting pattern in rice-crayfish farming system.

## 1. Introduction

The red swamp crayfish (*Procambarus clarkii*) belongs to Crustaceans, *Procambarus*, is one of the most important crustacean aquaculture species in China (The People's Republic of China Ministry of Agriculture, 2022). By 2021, the total production reached approximately 2.63 × 10^6^ tons. Integrated rice-crayfish farming model has developed vigorously in China, accounting for 80.77% of the farming area and contributed more than 83.54% of the total yield of crayfish in China (The People's Republic of China Ministry of Agriculture, 2022; XiuJuan et al., 2022).

Rice-crayfish system is a three-dimensional ecological agriculture model that effectively combines rice production and aquaculture. It guarantees a stable rice yield, significant raised the rice taste value and raised the economic benefits by 429.73% (Yang et al., 2021), and decreased CH_4_ emissions by 18.1–19.6%, decreased GWP by 16.8–22.0% (Sun Z. et al., 2019). Furthermore, the rice-crayfish system increased the soil total organic carbon, particulate organic carbon, strongly affects the soil microbial community composition, thus accelerating subsurface soil nutrient cycling (Si et al., 2017). Plants affect the composition of sediment microorganisms through root exudates (Bais et al., 2006; Orwin et al., 2006), and aquatic plants can form biofilms around the plant area to increase the microbial biomass of adjacent waters (Xie et al., 2011b). The transplanting pattern of rice affects the environmental parameters such as water and soil properties (Iqbal et al., 2014; Singh et al., 2020). Microbial composition in aquaculture environment not only interacts with the aquaculture environment parameters, but also affects growth and health of aquatic animals (Moriarty, 1997; Xie et al., 2011a; Lin et al., 2017; Hernandez-Perez et al., 2020). Different culture environments (pond or rice field) can significantly affect the relative abundance of intestinal microbial and archaeal communities of red swamp crayfish (Chen et al., 2021), and rice field model could provide a more stable intestinal environment and a better intestinal immune enzyme activity and muscular flavor (Liu et al., 2020).

Recently, several studies have indicated that sediment and water are major sources of intestinal microbes (Huang et al., 2014; Sun et al., 2020). More research shows that microbes in water is the main factor in determining the fitness of crustacean intestinal microbial communities. Rungrassamee et al. (2013) found that the initial intestinal microbe mainly came from the water, and the shrimp health status of ponds could be distinguishable and indicated by the bacterioplankton composition (Zhang et al., 2014). Based on these results, we hypothesize that different rice transplanting patterns may affect crayfish by influencing the environmental microorganisms. Intestinal microbiota in crustations were recognized as a key element for maintaining homeostasis and health, and numerous studies have shown that the intestinal microbiota appears to predict shrimp health status (Xiong et al., 2014; Dai et al., 2017). In fact, many intestinal microbial species participate in the digestion of food, they secrete some active substances, such as vitamins, essential amino acids, and highly unsaturated fat acids, which can be used by host (Almansa et al., 2012). In addition, crustaceans lack specific immunity and antibody mediated immune response, thus intestinal microbiota also affects the processes of immune response to maintain the normal function of the immune system (Vazquez et al., 2009; Musthaq and Kwang, 2014).

The transplanting environment of rice fields is crucial to the development of the crayfish farming industry (Moriarty, 1997; Abid et al., 2013; Cahenzli et al., 2013; Hou et al., 2014). Therefore, in this study, the high-throughput sequencing technology was used to explore the responses of microbial communities in water, sediment, and intestine to different rice transplanting patterns. This work aimed to investigate the responses of intestinal microbiota to different rice transplanting patterns, and determine the suitable rice cultivation pattern in rice-crayfish system.

## 2. Materials and methods

### 2.1. Sample collection

Water, sediment, and intestine samples were collected from nine transplanting fields (three experimental groups with three replicates in each group) located at Quanjiao district, Anhui Province, China (31.93° N, 118.18° E) on 26 May 2020 (Late tillering stage of rice). All fields had the same water source and shared the intake system. Each field had an area of approximately 2.7–2.8 ha. Rice and aquatic plants were planted simultaneously in the three groups in early April 2020. Each transplanting field had a circular trench around it, with a depth of approximately 1 m, and the area of ditch did not exceed 10% of the total plot area. Three ponds (PC) were planted with aquatic plants (*Elodea nuttallii*) in the field platforms. Three fields (RC) were planted with rice in the field platforms, and the spacing of rice was 0.2 m. Three fields (RC-W) were planted with rice in the field platforms, and the spacing of rice was 0.2 m and 0.4 m, respectively. During the farming period, crayfish formula feed was fed at 8:00 and 18:00 every day. Eight intestinal samples (pond culture GPC1-8, rice crayfish culture GRC1-8 and rice crayfish wide-narrow row planting GRC-W1-8), ten water samples (pond culture Verrucomicrobiaceae 1–10, rice crayfish culture WRC1-10 and rice crayfish wide-narrow row planting WRC-W1-10), and nine sediment samples (pond culture SPC1-9, rice crayfish culture SRC1-9 and rice crayfish wide-narrow row planting SRC-W1-9) were taken from three fields for each group. Water samples were randomly collected from 9 fields using a glass water hydrophore. The water sample (50 mL) was filtered using a polycarbonate membrane (Millipore, USA) with a pore size of 0.22 μm. After filtration, the membrane was placed in a 2 mL centrifuge tube. Another part of water sample (500 mL) was collected to measure water quality. The water quality (nitrite, nitrate, ammonia, total nitrogen, phosphate and total phosphorus) was measured by using standard analytical methods. Dissolved oxygen, and pH were measured by using a Hach HQ40d Portable Multi-probe Multi Handheld Meter (USA). Water quality indicators denote significant differences as evaluated by Tukey’s HSD test (*p* < 0.05). Sediment samples were random collected from 9 fields using a core sampler and each sediment sample (2–3 cm) was placed in a 5 mL sterile centrifuge tube. The surfaces of the crayfishes were sterilized with 70% ethanol. The intestines were aseptically separation and placed in a sterile 1.5 mL centrifuge tube, all samples were stored at −80°C immediately before DNA extraction.

### 2.2. DNA extraction and sequencing

Total DNA of water filter membranes, sediment and intestinal samples were extracted following the E.Z.N.A.® Water (Soil, Stool) DNA Kit (Omega Bio-tek, Norcross, GA, U.S.) according to the manufacturer’s protocols. PCR amplification of the bacterial 16S rRNA genes V3-V4 region was performed using the forward primer 338F (5’-ACTCCTACGGGAGGCAGCA-3′) and the reverse primer 806R (5’-GGACTACHVGGGTWTCTAAT-3′). Sample-specific 7-bp barcodes were incorporated into the primers for multiplex sequencing. The PCR components contained 5 μL of Q5 reaction buffer (5×), 5 μL of Q5 High-Fidelity GC buffer (5×), 0.25 μL of Q5 High-Fidelity DNA Polymerase (5 U/μl), 2 μL (2.5 uM) of dNTPs, 1 μL (10 uM) of each forward and reverse primer, 2 μL of DNA Template, and 8.75 μL of ddH2O. Thermal cycling consisted of initial denaturation at 98°C for 2 min, followed by 25 cycles consisting of denaturation at 98°C for 15 s, annealing at 55°C for 30 s, and extension at 72°C for 30 s, with a final extension of 5 min at 72°C. PCR amplicons were purified with Agencourt AMPure Beads (Beckman Coulter, Indianapolis, IN) and quantified using the PicoGreen dsDNA Assay Kit (Invitrogen, Carlsbad, CA, USA). After the individual quantification step, amplicons were pooled in equal amounts, and pair-end 2 × 300 bp sequencing was performed using the Illlumina MiSeq platform with MiSeq Reagent Kit v3 at Shanghai Personal Biotechnology Co., Ltd. (Shanghai, China).

### 2.3. Bioinformatics and statistical analysis

The Quantitative Insights Into Microbial Ecology (QIIME, v1.9.1) pipeline was employed to process the sequencing data, as previously described (Caporaso et al., 2010). Briefly, raw sequencing reads with exact matches to the barcodes were assigned to respective samples and identified as valid sequences. The low-quality sequences were filtered through following criteria (Gill et al., 2006; Chen and Jiang, 2014) sequences that had a length of <150 bp, sequences that had average Phred scores of <20, sequences that contained ambiguous bases, and sequences that contained mononucleotide repeats of >8 bp. Paired-end reads were assembled using FLASH (Magoc and Salzberg, 2011). After chimera detection, the remaining high-quality sequences were clustered into operational taxonomic units (OTUs) at 97% sequence identity by UCLUST (Edgar, 2010). A representative sequence was selected from each OTU using default parameters. OTU taxonomic classification was conducted by BLAST searching the representative sequences set against the Greengenes Database (DeSantis et al., 2006) using the best hit (Altschul et al., 1997).

The diversity index was calculated from the OTUs of each library to estimate and compare the microbial community diversity in each group. Welch’s t-test was used to compare the microbial diversity and OTU richness of water, sediment, and crayfish intestine; *p* < 0.05 was considered significant. A Principal Component Analysis (PCA), Network analysis (Networkx), Redundancy analysis (RDA) and Venn diagrams were carried out in the R environment (version 3.3.1), explored and visualized using the Majorbio I-Sanger Cloud Platform.^1^ Raw reads were deposited in the NCBI Sequence Read Archive (SRA) database Accession Number: SUB12421940.

## 3. Results

### 3.1. Status of water quality parameters

Table 1 shows the results of water quality monitoring across the 9 fields. By comparing the results, it can be seen that water physical–chemical properties varied greatly under different rice planting patterns. The dissolved oxygen values varied from 5.73 to 11.28 mg/L (Table 1) in different groups, whereby the highest value was recorded in WRC. The labile Phosphorus in this study varied from 1.42 to 2.53 μg/L (Table 1), with the highest levels in WRC and the lowest levels in WPC. The ANOVA test showed that there were significant differences (*p* < 0.05) among the three groups.

**Table 1:** Means of the water physical and chemical parameters in the three habitats.

	SRP (ug/l)	NO_2_^−^-N (ug/l)	NO_3_^−^-N (ug/l)	NH_4_^+^-N (mg/l)	TP (mg/l)	TN (mg/l)	DO (mg/l)	pH
WPC	1.42 ± 0.13^a^	14.35 ± 0.38	39.45 ± 3.09	0.05 ± 0.02	0.12 ± 0.01^a^	3.26 ± 0.13	5.73 ± 0.43^a^	7.76 ± 0.15
WRC-W	1.53 ± 0.22^a^	14.43 ± 0.57	32.57 ± 4.65	0.08 ± 0.04	0.14 ± 0.01^b^	3.31 ± 0.19	8.21 ± 1.44^b^	7.63 ± 0.14
WRC	2.53 ± 0.86^b^	13.95 ± 0.53	33.55 ± 3.3	0.05 ± 0.02	0.12 ± 0.02^c^	3.58 ± 0.36	11.28 ± 3.01^b^	7.35 ± 0.13

Water-associated physicochemical factors. SRP, labile phosphate; NO_2_^−^-N, nitrite nitrogen; NO_3_^−^-N, nitrate nitrogen; NH_4_^+^-N, ammonium nitrogen; TP, total phosphorus; TN, total nitrogen; DO, dissolved oxygen; pH. Different superscript letters within a list denote significant differences as evaluated by Tukey’s HSD test (*p* < 0.05).

### 3.2. Characteristics of 16S rRNA sequencing and microbial community diversity

A total of 4,772,094 reads were obtained from high-throughput sequencing of the V3-V4 regions of 16S rRNA genes. The total number of OTUs were 3,326, 3,239 and 1,504 in water, sediment and intestine, respectively. The number of OTUs varied greatly among the three experimental groups. The diversity index was calculated from OTUs of each library to estimate and compare the microbial community diversity in each sample. Microbial community diversity estimated by Shannon’s index varied from 2.40 to 2.73 in intestines, 3.81 to 5.02 in water, 5.71 to 5.87 in sediment (Table 2). ACE analysis conducted for estimating microbial community richness indicated a range of values from 407.71 to 550.15 in intestines, 1268.6 to 1627.5 in water, and 1844.7 to 1905.6 in sediment (Table 2). These results indicated that, compared with water and sediment, crayfish intestine had the lowest OTU richness and microbial community diversity. Welch’s t-test result showed that there were significant differences in the community diversity and OTUs richness of the water (*p* < 0.001), the OTUs richness of the intestines were significant different (*p* < 0.001), while the microbial diversity and OTUs richness of sediment were not significantly different among the three experimental groups.

**Table 2:** Summary of the evenness index (Shannon) and estimated OTU richness (ACE) for the prokaryotic community diversity analysis from the intestinal, water, and sediment samples.

Estimators	WPC	WRC-W	WRC	GPC	GRC-W	GRC	SPC	SRC-W	SRC
ACE	1593.20^a^	1531.50^a^	1799.40 ^b^	558.02^a^	514.18^b^	692.47^c^	2067.60^a^	2111.40^a^	2125.10^a^
Shannon	5.00^a^	3.83^b^	5.07^a^	2.41^a^	2.54^a^	2.76^a^	5.91^a^	5.74^a^	5.85^a^

ACE, community richness; Shannon, community evenness. Different superscript letters within a line denote significant differences as evaluated by Welch’s t-test (*p* < 0.05).

### 3.3. Overall microbial communities in water, sediment, and crayfish intestine

**Figure 1:**
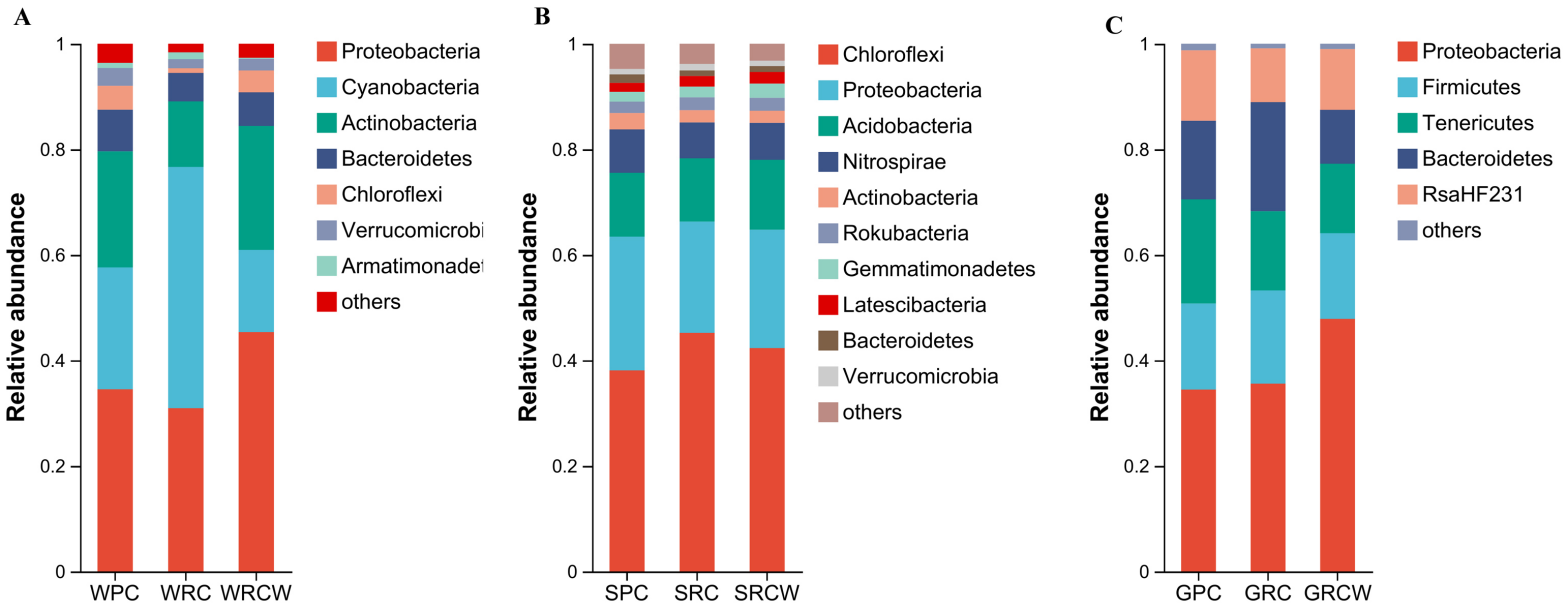
The phylum level relative abundance of microbial communities in the *Procambarus clarkii* intestines, surrounding water, and sediment samples. **(A)** Water microorganisms, **(B)** Sediment microorganisms, **(C)** Intestinal microbiota The abscissa is the group name, and the ordinate is the phylum name. Only phyla that are present at relative abundance >1% in at least one sample are shown.

**Figure 2:**
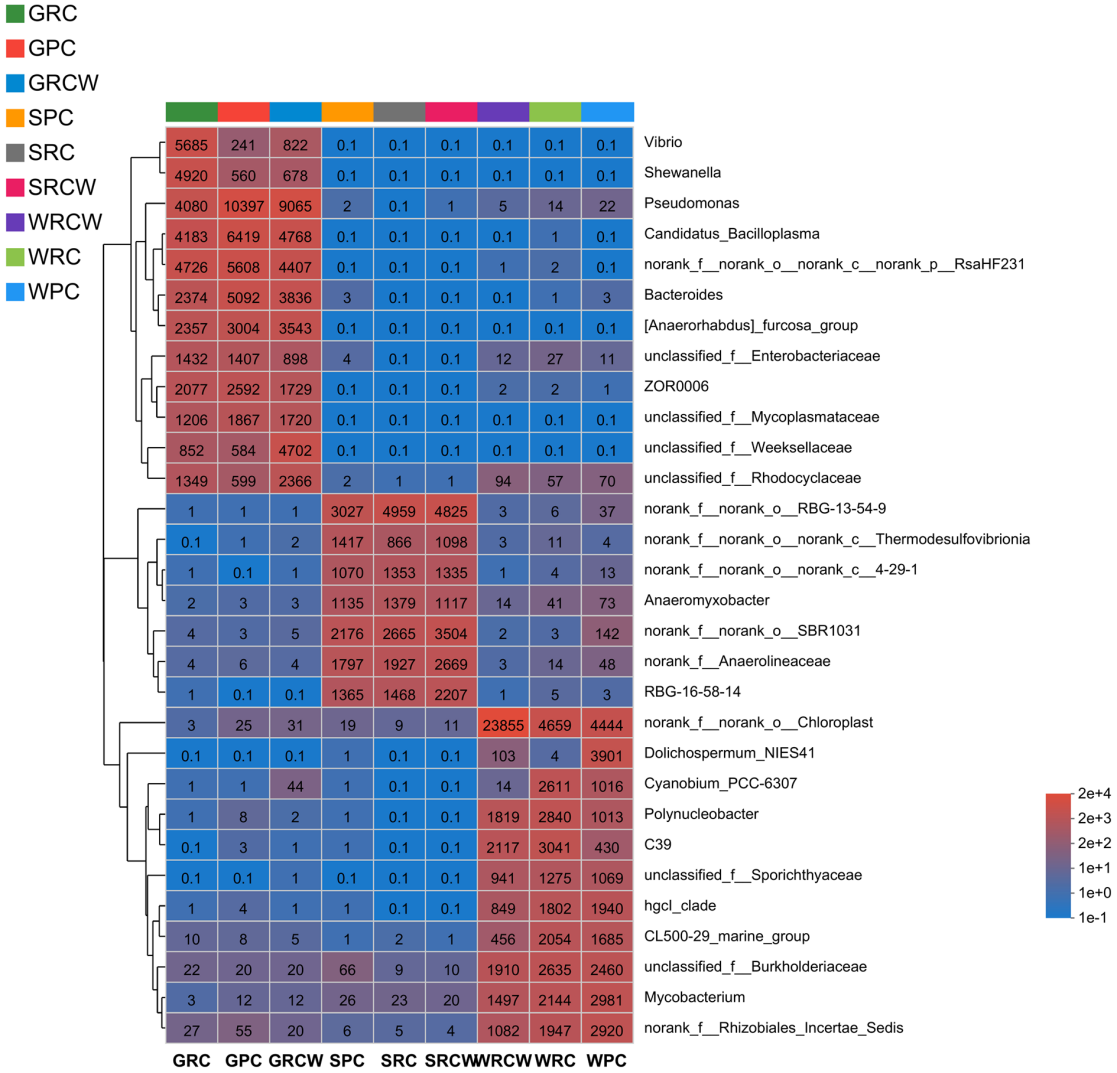
The genus level relative abundance of bacterial communities in the crayfish intestines, water, and sediment samples. The abundance changes of different species in the sample are displayed through the color gradient (number of OTUs) of the color block.

Figures 1, 2 shows the OTU distribution of 78 samples, including 3,239 sediment OTUs, 3,326 water OTUs and 1,504 intestinal OTUs. A total of 15 microbial phyla (OTU values >1%) were detected as shown in Figure 1. Figure 1 shows the abundant phyla in the intestine, water and sediment samples. Proteobacteria, Chloroflexi, and Proteobacteria had the highest relative abundance in intestine, water, and sediment samples, respectively. The Proteobacteria relative abundance fluctuated around 34.47% for GPC, 35.60% for GRC-W, 47.87% for GRC, 34.50% for WPC, and 34.50% for WRC-W for 30.93, 45.36% for WRC. The Chloroflexi relative abundance fluctuated around SPC for 38.10%, SRC-W for 45.23% and SRC for 42.34% (Figure 1).

**Figure 3:**
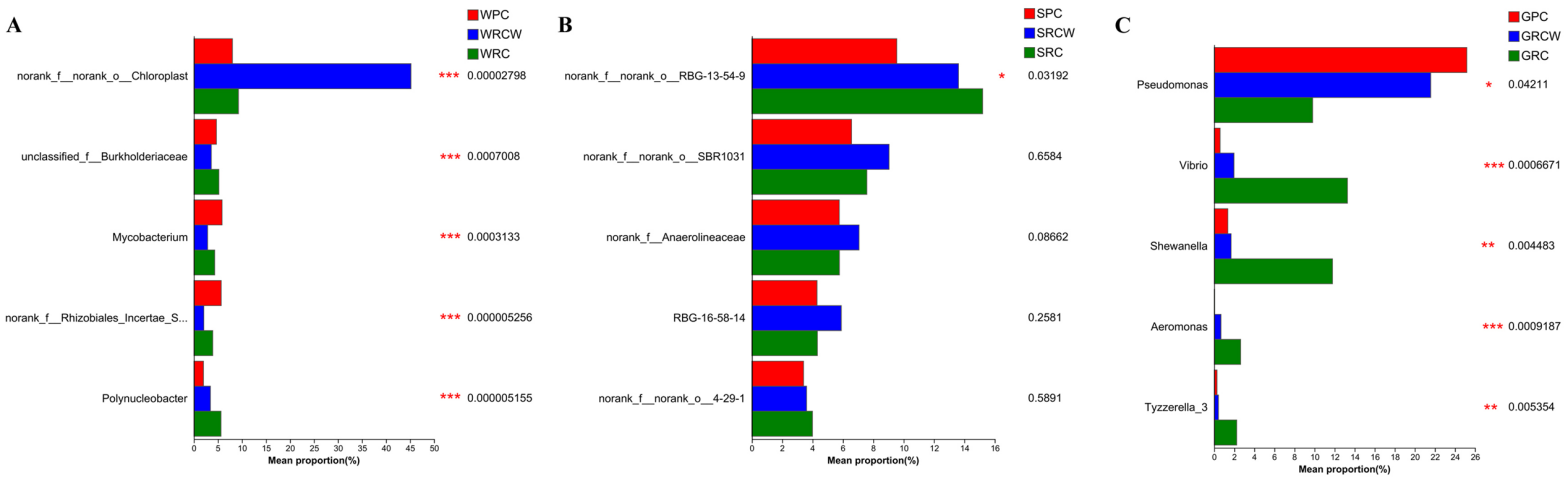
Test for statistical significance in the intergroup rank-sum in the intestines, water, and sediment samples. The value represented by the color gradient is shown on the right side of the figure. Columns of different colors represent different groups, and the rightmost point is the *p*-value. **(A)** Water microorganisms, **(B)** Sediment microorganisms, **(C)** intestinal microbiota.

The composition of dominant taxa of each sample at the genus levels can be seen in Figure 2. The dominant bacterial genus in intestine samples were *Pseudomonas, Candidatus_Bacilloplasma, Rashf231, Bacteroides, Anaerorhabdu_Furcosa_Group, Vibrio, ZOR0006, Shewanella*. The dominant bacterial genus of water samples was *norank_o_chloroplast, unfied_burkhoideraceae, Mycobacterium, norank_f_rhizobiales_incertae_sedfis, polynucleobacter, C39, hgcl_clade, CL500-29_marine_group*. The dominant bacterial genus in sediment samples was *norank_f_norank_o_RBG-13-54-9, norank_f_norank_o_SBR1031, norank_f_anaerolineaceae, RBG-16-58-14, norank_f_norank_o_noranka_c_kd4–29-1, norank_f_norank_o_noranka_c_KD4-96, Anaeromyxobacer*. As seen in Figure 3, genus classification revealed that samples differed in terms of dominant microbial genera (*p* < 0.05). There were significant differences of dominant microbial (top 5 genus) in water and intestinal samples, which *norank_o_RBG-13-54-9* presented differed significant in soil samples.

**Figure 4:**
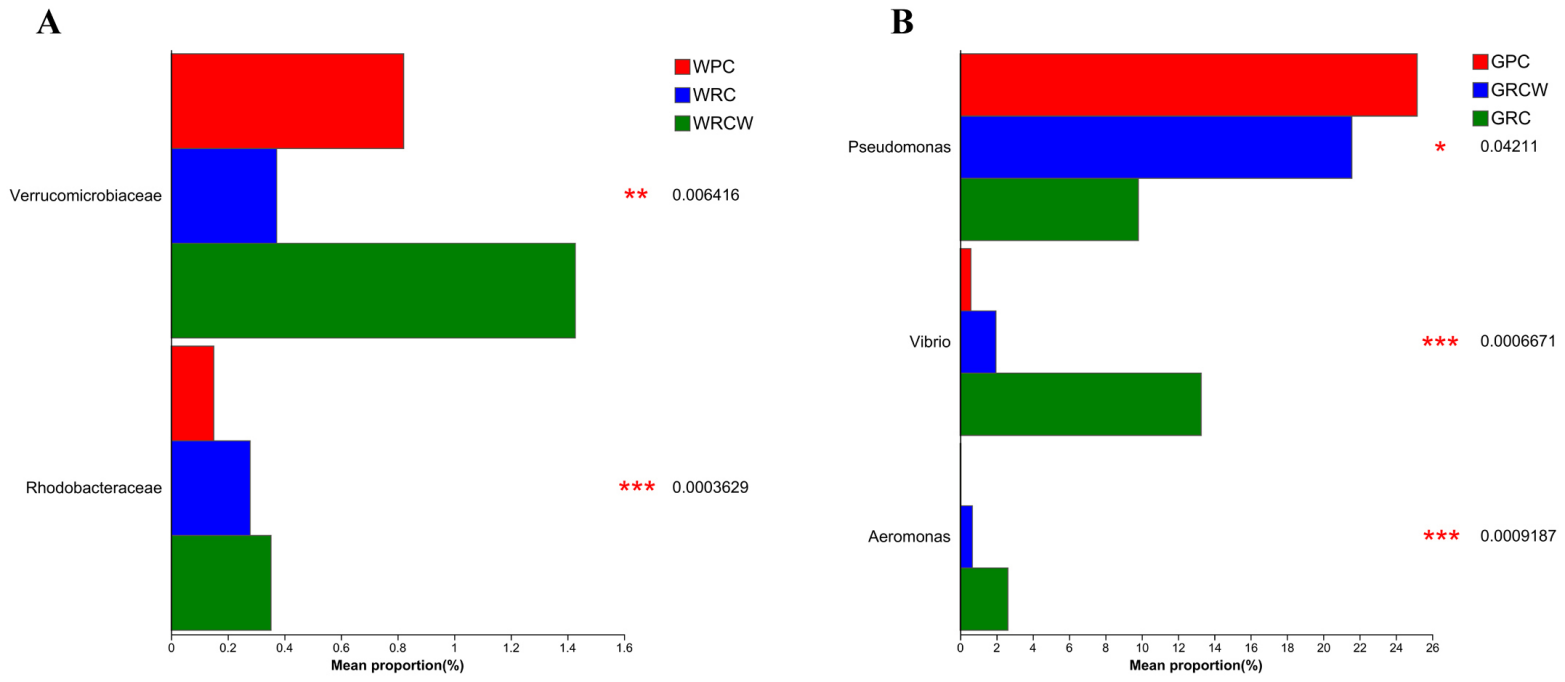
The relative abundance of two types of aquatic microorganisms known for their indicative value and three common harmful bacteria. **(A)** Rhodobacteraceae and Verrucomicrobiaceae were microorganisms in water bodies, **(B)** *Pseudomonas*, *Vibrio*, *Aeromonas* were intestinal microbiota.

In order to assess the environmental health of the aquaculture systems, two types of aquatic microorganisms known for their indicative value and three categories of bacteria commonly associated with infections in aquatic organisms were selected for analysis. The findings revealed noteworthy differences in abundance among these microorganisms. Specifically, in terms of water samples, the relative abundance of Rhodobacteraceae was significantly lower in both the RC-W group and PC group compared to the RC group. Furthermore, the relative abundance of Verrucomicrobiaceae in the RC-W group was notably lower compared to the other two groups. When considering intestinal microbiota, the relative abundance of *Vibrio* and *Aeromonas* in the RC-W group exhibited significantly higher levels compared to the other two groups. Conversely, the relative abundance of *Pseudomonas* in the RC-W group was significantly lower when compared to the other two groups (Figure 4).

### 3.4. Correlation between intestine and environmental microbe in the three experimental groups

**Figure 5:**
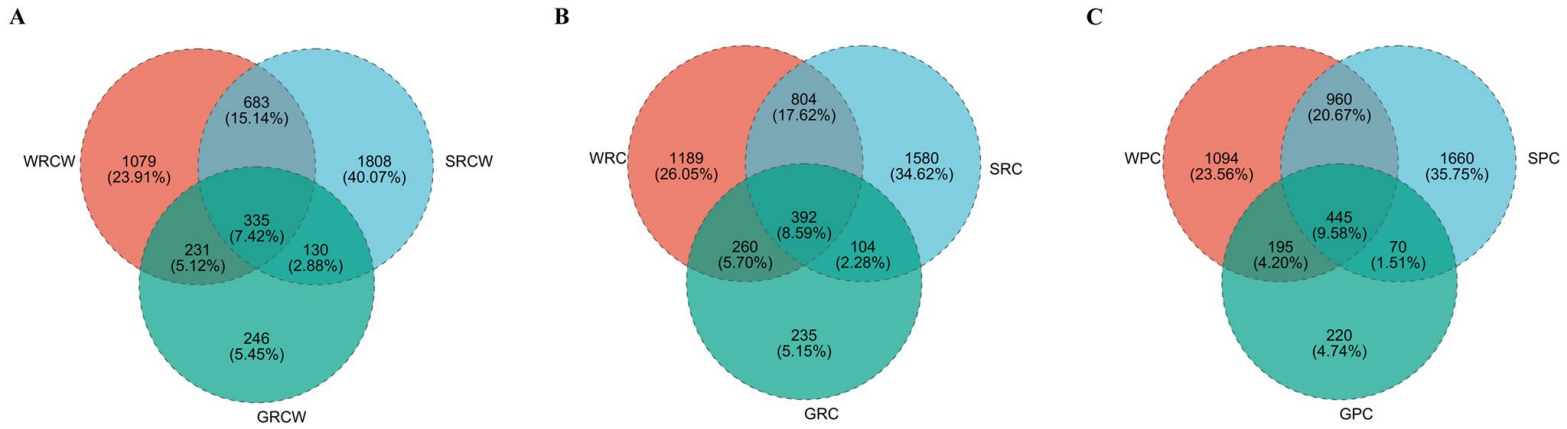
Venn diagram analysis of the OTUs numbers in the crayfish intestine **(A)** sediment **(B)**, and water **(C)** samples. Different colors represent different groups, overlapping numbers represent the number of species common to multiple groups, and nonoverlapping numbers represent the number of species unique to the corresponding group.

In the PC group, the Venn shows the number of intestinal OTUs shared with sediment and water accounted for 445. In the RC-W group, the Venn shows the number of intestinal OTUs shared with sediment and water accounted for 335. In the RC group, the Venn shows the number of intestinal OTUs shared with sediment and water accounted for 392 (Figure 5). The correlation analysis was performed on the species abundances of water, sediment and intestine microbes in the three groups by Networkx software. The results showed that the bacteria (top 30 genera) in the three groups shared a high-er number of populations in the water and intestinal samples than in the sediment samples. *Pseudomonas* dominated the PC group and the RC-W group, which *Vibrio* dominated the RC group (Table 3).

**Table 3:** Attributes of the water, sediment, and intestinal microbial network nodes in the three experimental groups.

Group ID	Node name	Weighted degree
PC	*g__Pseudomonas*	10,421.13611
PC	*g__Candidatus_Bacilloplasma*	6,418.975
PC	*g__norank_f__norank_o__norank_c__norank_p__RsaHF231*	5,607.85
PC	*g__Bacteroides*	5,097
RC-W	*g__Pseudomonas*	9,070.59167
RC-W	*g__norank_f__norank_o__RBG-13-54-9*	4,828.82778
RC-W	*g__Candidatus_Bacilloplasma*	4,768.775
RC-W	*g__unclassified_f__Weeksellaceae*	4,702.475
RC	*g__Vibrio*	5,685.125
RC	*g__norank_f__norank_o__RBG-13-54-9*	4,966.76944
RC	*g__Shewanella*	4,919.875
RC	*g__norank_f__norank_o__norank_c__norank_p__RsaHF231*	4,727.5

**Figure 6:**
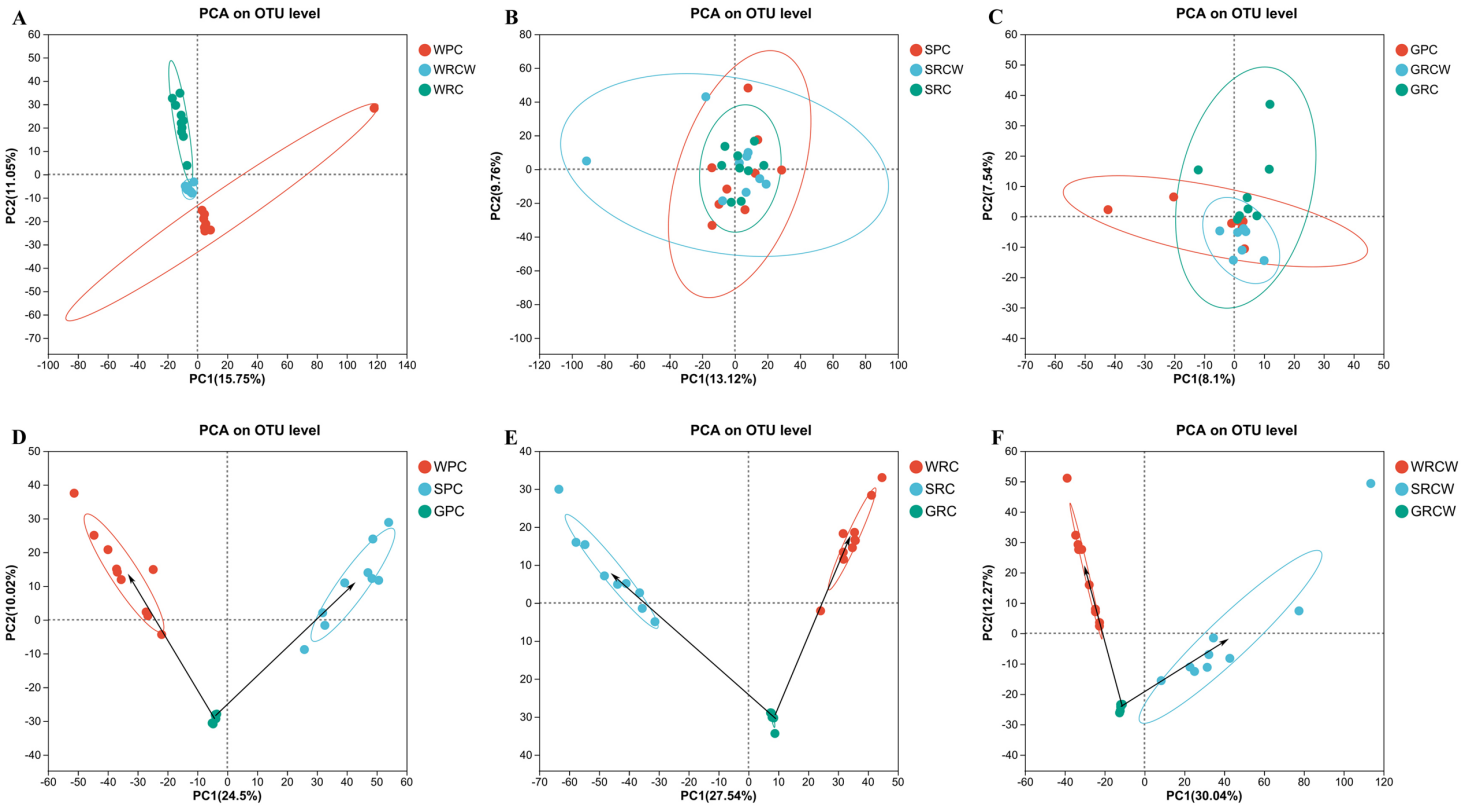
Similarity among the microbial communities associated with different samples. Principal Component Analysis (PCA) based on weighted UniFrac analysis of microbe. Points of different colors represent samples of different groups, and distance between sampling points represents the level of similarity. **(A)** Water microorganisms, **(B)** Sediment microorganisms, **(C)** Intestinal microbiota, **(D)** Pond-crayfish, **(E)** Rice-crayfish with normal transplanting, **(F)** Rice crayfish with wide-narrow row transplanting.

The similarity matrix of the samples was analyzed by PCA. All samples of the same group (source) tended to cluster together in Figure 6. The sediment samples showed the highest similarity and shortest distance between each other, no significant differences in microbial communities among three groups. Some intestine samples showed a high level of variability. In the three groups, the biological distance of microbial communities in water samples were closer to that of the intestinal samples of *Procambarus clarkii* than that of the sediment samples.

**Figure 7:**
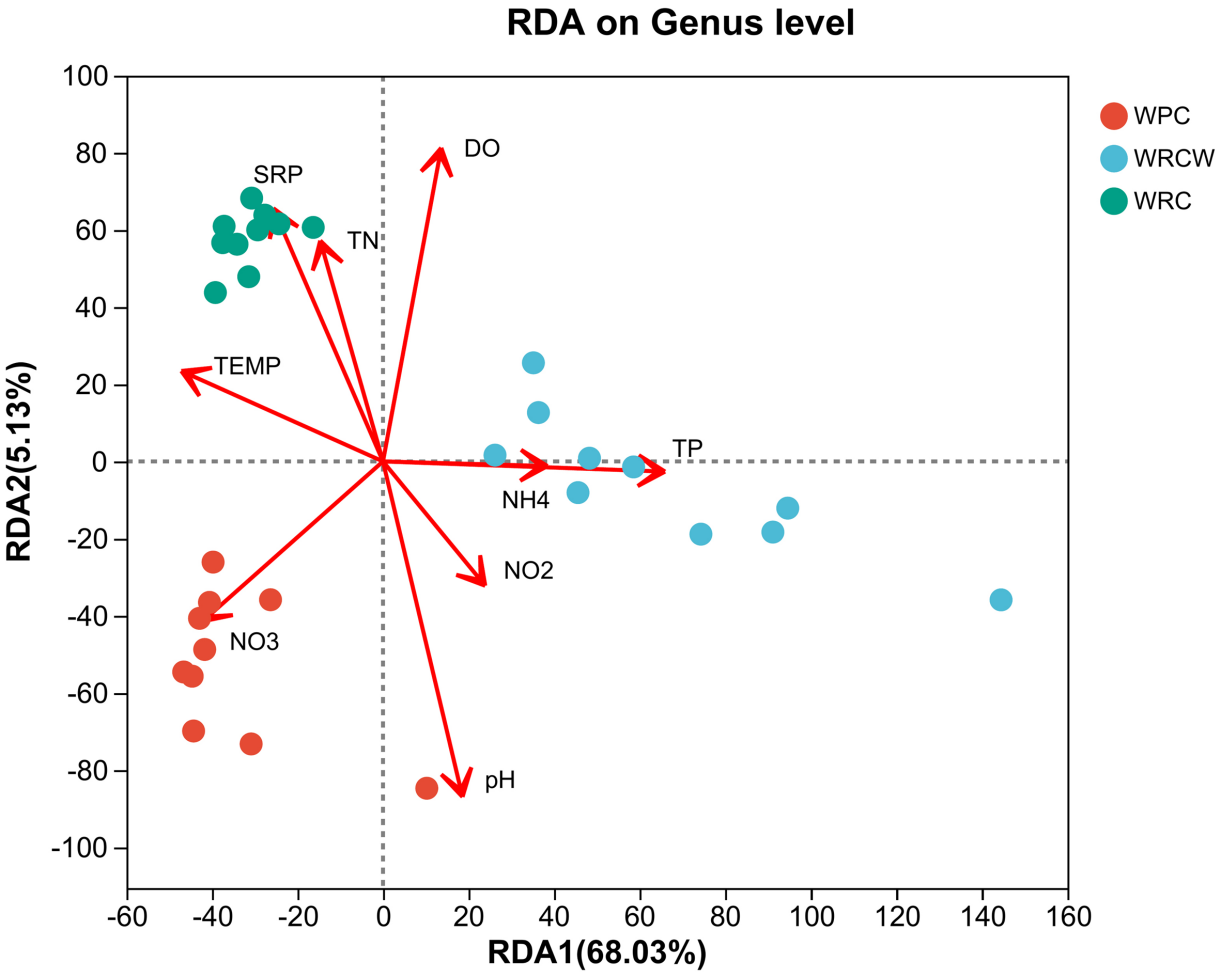
Redundancy analysis (RDA) of the dominant water microbial taxa and the environmental factors. The dots with different colors in the figure represent sample groups in different environments. The length of the environmental factor arrow can represent the degree of impact of environmental factors on species data; The angle between the arrows of environmental factors represents a positive and negative correlation.

RDA was used to identify the key relationship of environmental factors with microbial community. Figure 7 shows a clear correlation between the microbial communities in water and water environmental parameters, namely, pH, DO, nitrate, and the intestinal microbial community and water environmental parameters, namely, Total nitrogen, pH, total phosphorus, phosphate, nitrate. *Pseudomonas* had a significant positive correlation with nitrate, nitrite, temperature, pH, and negatively correlated with total nitrogen, total phosphorus, phosphate. Pathogenic bacteria, including *Vibrio*, *Shewanella* had a significant positive correlation with phosphate, total nitrogen, temperature, DO, and negatively correlated with nitrate, nitrite, total phosphorus, pH.

## 4. Discussion

Understanding the impacts of rice transplanting patterns on a rice–crayfish system’s environment is crucial as the aquaculture environment is closely related to aquatic animals, which has been widely studied (Cahenzli et al., 2013; Sun F. et al., 2019; Sun et al., 2020). Therefore, this study used high-throughput sequencing to analyze the microbial community compositions of intestinal, water, and sediment samples under different rice transplanting patterns in rice–crayfish systems. It was found that rice transplanting patterns significantly changed the biodiversity and species composition of the water and intestinal samples, particularly, the levels of *Procambarus clarkii*.

These results indicated that how the rice was transplanted had significant effects on the microbial community structures of the water bodies. The relative abundance of the dominant phyla and genera showed significant differences among the three groups, and the PCA results proved that the three groups were highly differentiated. The dominant bacterial phyla recorded in the water were Proteobacteria, Cyanobacteria, Actinobacteria, Bacteroidetes, which are the most common microflora in an aquaculture environment (Rungrassamee et al., 2013; Sun F. et al., 2019). Environmental factors could directly alter microbial community structures by inhibiting microbial physiology, or indirectly by producing conditions that affect the microorganisms (Fuhrman et al., 2008; Lin et al., 2017). Rice transplanting patterns may affect the microbial community composition of water by affecting the physical and chemical properties of the water in fields (Orwin et al., 2006; Panizzon et al., 2013). In the present study, using redundancy analysis, the pH, and DO levels were found to be the primary parameters affecting the microbial communities in water, which was consistent with previous studies on water microorganisms (Addo et al., 2021; Li et al., 2021). The oxygen provided by plants to the microbes is an important aspect of the mutually beneficial relationship between plant and microbes (Srivastava et al., 2016), and it may be the reason why the microbial community richness in water of the RC group was significantly higher than that of the PC and RC-W groups. An interesting finding in this study was that the relative abundance of Cyanophyta in the paddy water with a conventional transplanting pattern was significantly lower than that of the other two groups. Cyanobacteria outbreaks often pose a threat to aquaculture production by affecting the water quality, community structure, and the survival of economic animals (Vasconcelos and Pereira, 2001; Romo et al., 2012). As light is one of the limiting conditions for the growth of Cyanobacteria (Reynolds, 2006; Foy et al., 2007), it is speculated that the shading effect of rice plants on sunlight is the primary reason to restrict the growth of Cyanobacteria.

Different rice transplanting patterns had no significant effects on sediment microbial biodiversity. A large number of studies have proven that soil type is the primary influencing factor on sediment microorganisms (Chiarini et al., 1998; Gelsomino et al., 1999; Silva et al., 2003). In this study, each experimental plot was adjacent, and their soil types were consistent. This could explain why there were no significant differences in sediment microbial biodiversity in the different rice transplanting patterns or even in the control group (the pond model). However, the presence of rice in a field continues to impact the composition of sediment microorganisms. Existing studies have shown that the type and quantity of vegetation are the primary factors affecting sediment microorganisms (Iii et al., 2009; Gray et al., 2011). Among the three experimental groups, the relative abundance of Planctomycetes and Firmicutes in the fields without rice (PC) was significantly higher than that of the fields with rice (RC and RC-W), and relevant studies have confirmed that crops could significantly reduce the relative abundance of Planctomycetes and Firmicutes in sediment (Zhang et al., 2020).

The specificity of the microbial communities in the intestines was regulated by the selective pressure of the intestinal habitat and the host genotype (Rawls et al., 2006; Rungrassamee et al., 2014). While previous studies have shown that most crustaceans have a relatively stable core microorganisms in their intestines (Sun F. et al., 2019; Sun et al., 2020), the OTUs shared by intestinal microbes in the three groups accounted for only 27.21% of the total OTUs, indicating that environmental microbes have important effect on the intestinal microbes of *Procambarus clarkii*. In this study, the intestinal community richness of *Procambarus clarkii* in the RC group was significantly higher than those of the RC-W and PC groups, which was positively correlated with the community richness in the water. The closer biological distance revealed by the principal component analysis further suggested that the microbial communities in water samples may be the primary source of intestinal samples. At the genus level, we found that that the relative abundance of *Aeromonas* in the intestinal samples was positively correlated with that in the water samples, which further confirmed water as the primary source of intestinal microbiota. Previous studies have shown that *Aeromonas* species in the intestines are affected by environmental elements (Caruso et al., 2004; Rungrassamee et al., 2013), and water microbes were the primary influencing factors on the intestinal microbial composition (Rungrassamee et al., 2013; Sun F. et al., 2019). Therefore, the absence of rice in the field also affected the intestinal microbiota of the *Procambarus clarkii*. In the PC group, the *Procambarus clarkii* fed on more aquatic plants than those of the RC and RC-W groups, and Bacteroidetes could ferment the plant-derived substrates in the intestines, enabling the hosts to obtain additional energy (Nayak, 2010; van Rooyen et al., 2011), which could better explain the relative abundance of Bacteroidetes in the intestines of the *Procambarus clarkii* in the PC group, which was significantly higher than that of the other two groups.

In intestinal microecosystems, each bacterium occupies a specific ecological niche (Cadotte, 2004), and susceptibility to invasion by exotic species is strongly influenced by species composition and generally decreases with increasing species richness (Hooper et al., 2005). Previous studies have shown that intestinal microbial biodiversity in healthy aquatic animals is often higher than that of in diseased animals (Liu et al., 2018). Therefore, we hypothesized that the intestinal microecosystem of *Procambarus clarkii* in paddy fields was more resistant to external pathogens. From the perspective of microbial biodiversity, the higher microbial biodiversity of the water in the RC group implied that a water ecosystem may be more stable under a normal rice transplanting pattern (Tilman et al., 2001; Hooper et al., 2012). However, the results were not as expected from the perspective of the abundance of some disease-associated microbial groups. Previous studies have shown that the relative abundance of Rhodobacteraceae and Verrucomicrobiaceae in pathogenic shrimp ponds was significantly higher than that of healthy ponds (Zhang et al., 2014). The lower relative abundance of Rhodobacteraceae and Verrucomicrobiaceae suggested that the RC-W group appeared to have a healthier environment. The relative abundance of *Vibrio* and *Aeromonas*, which are the primary pathogens of aquatic animals (Zeng, 2020; Gan et al., 2022), was higher in the RC group among the three groups of intestinal microorganisms. The numbers and types of pathogenic bacteria are the key factors affecting the production of high-quality aquatic products, and these bacteria are directly affected by environmental factors (Caruso et al., 2004; Anneke et al., 2013). Therefore, in terms of the abundance of pathogenic microorganisms, narrow and wide transplanting patterns may be better choices for rice–crayfish farming plots.

In conclusion, the different rice transplanting patterns had significant effects on microbial biodiversity and species composition in surrounding water. Under the conditions of this experiment, the intestinal community richness of *Procambarus clarkii* in the three groups was positively correlated with the community richness of the water. The proximity between intestinal and water samples in the PCA diagram indicated that their species composition was more similar. Rice transplanting patterns can affect the intestinal microbial biodiversity of *Procambarus clarkii*, and the water microbes were the primary factor affecting the intestinal microbes. Although the diversity of environmental microorganisms was better under conventional rice planting patterns, from the perspective of pathogenic microorganisms, a wide–narrow row transplanting pattern may be a more beneficial choice for rice–crayfish farming.

## Data availability statement

The datasets presented in this study can be found in online repositories. The names of the repository/repositories and accession number(s) can be found at: https://www.ncbi.nlm.nih.gov/, SUB12421940.

## Ethics statement

All experiments were performed according to the Experimental Animal Management Law of China and approved by the Animal Ethics Committee of Shanghai Ocean University. The studies were conducted in accordance with the local legislation and institutional requirements. Written informed consent was obtained from the owners for the participation of their animals in this study.

## Author contributions

JH drafted the manuscript and participated in the experiment. JH and JinL performed sample preparation. JiaL and WZ helped to analyzed data. YC interpreted results and sample preparation. JH and JiaL conceived and designed the study. All authors read and gave final approval of the final manuscript.

## Funding

This study was funded by the Science and Technology Project of Social Development of the Shanghai Municipal Science and Technology Commission with grant number (21DZ1201900), the National Key Research and Development Program of China with grant number (2019YFD0900304) and the earmarked fund for China Agriculture Research System (CARS) (CARS-48).

## Conflict of interest

The authors declare that the research was conducted in the absence of any commercial or financial relationships that could be construed as a potential conflict of interest.

The reviewer ZY declared a past co-authorship with the author YC to the handling editor.

## Publisher’s note

All claims expressed in this article are solely those of the authors and do not necessarily represent those of their affiliated organizations, or those of the publisher, the editors and the reviewers. Any product that may be evaluated in this article, or claim that may be made by its manufacturer, is not guaranteed or endorsed by the publisher.

## Footnotes

^1^https://www.majorbio.com

## References

[ref1] Abid A. Davies S. J. Waines P. Emery M. Castex M. Gioacchini G.. (2013). Dietary synbiotic application modulates Atlantic salmon (*Salmo salar*) intestinal microbial communities and intestinal immunity. Fish Shellfish Immunol. 35, 1948–1956. doi: 10.1016/j.fsi.2013.09.039, PMID: 24161776

[ref2] Addo F. G. Zhang S. Manirakiza B. Ohore O. E. Shudong Y. (2021). The impacts of straw substrate on biofloc formation, bacterial community and nutrient removal in shrimp ponds. Bioresour. Technol. 326:124727. doi: 10.1016/j.biortech.2021.124727, PMID: 33548819

[ref3] Almansa C. Agrawal A. Houghton L. A. (2012). Intestinal microbiota, pathophysiology and translation to probiotic use in patients with irritable bowel syndrome. Expert Rev. Gastroenterol. Hepatol. 6, 383–398. doi: 10.1586/EGH.12.9, PMID: 22646259

[ref4] Altschul S. F. Madden T. L. Schaffer A. A. Zhang J. H. Zhang Z. Miller W.. (1997). Gapped BLAST and PSI-BLAST: a new generation of protein database search programs. Nucleic Acids Res. 25, 3389–3402. doi: 10.1093/nar/25.17.3389, PMID: 9254694 PMC146917

[ref5] Anneke E. Lenny H. Jan S. (2013). Pathogen-host-environment interplay and disease emergence. Emerg. Microbes Infect. 2:e5. doi: 10.1038/emi.2013.5, PMID: 26038452 PMC3630490

[ref6] Bais H. P. Weir T. L. Perry L. G. Gilroy S. Vivanco J. M. (2006). The role of root exudates in rhizosphere interactions with plants and other organisms. Annu. Rev. Plant Biol. 57, 233–266. doi: 10.1146/annurev.arplant.57.032905.105159, PMID: 16669762

[ref7] Cadotte M. (2004). Review of ecological niches: linking classical and contemporary approaches, by J. M. Chase and M. A. Leibold. Biodiver. Conserv. 13, 1791–1793. doi: 10.1023/b:bioc.0000029366.24837.fc

[ref8] Cahenzli J. Balmer M. L. Mccoy K. D. (2013). Microbial–immune cross-talk and regulation of the immune system. Immunology 138, 12–22. doi: 10.1111/j.1365-2567.2012.03624.x, PMID: 22804726 PMC3533697

[ref9] Caporaso J. G. Kuczynski J. Stombaugh J. Bittinger K. (2010). QIIME allows analysis of high-throughput community sequencing data. Nat. Methods 7, 335–336. doi: 10.1038/nmeth.f.303, PMID: 20383131 PMC3156573

[ref10] Caruso G. Maimone G. Mancuso M. Modica A. Genovese L. (2004). Microbiological controls across the productive cycle of *Dicentrarchus labrax* L. and *Sparus aurata* L.: a study from the environment to the final product. Aquac. Res. 35, 184–193. doi: 10.1111/j.1365-2109.2004.01009.x

[ref11] Chen X. Fan L. M. Qiu L. P. Dong X. X. Wang Q. Hu G. D.. (2021). Metagenomics analysis reveals compositional and functional differences in the gut microbiota of red swamp crayfish, *Procambarus clarkii*, grown on two different culture environments. Front. Microbiol. 12:5190. doi: 10.3389/fmicb.2021.735190, PMID: 34733252 PMC8558459

[ref12] Chen H. Jiang W. (2014). Application of high-throughput sequencing in understanding human oral microbiome related with health and disease. Front. Microbiol. 5:508. doi: 10.3389/fmicb.2014.00508, PMID: 25352835 PMC4195358

[ref13] Chiarini L. Bevivino A. Dalmastri C. Nacamulli C. Tabacchioni S. (1998). Influence of plant development, cultivar and soil type on microbial colonization of maize roots. Appl. Soil Ecol. 8, 11–18. doi: 10.1016/S0929-1393(97)00071-1

[ref14] Dai W. F. Yu W. N. Zhang J. J. Zhu J. Y. Tao Z. Xiong J. B. (2017). The gut eukaryotic microbiota influences the growth performance among cohabitating shrimp. Appl. Microbiol. Biotechnol. 101, 6447–6457. doi: 10.1007/s00253-017-8388-0, PMID: 28702793

[ref15] Desantis T. Z. Hugenholtz P. Larsen N. Rojas M. Brodie E. L. Keller K.. (2006). Greengenes, a chimera-checked 16S rRNA gene database and workbench compatible with ARB. Appl. Environ. Microbiol. 72, 5069–5072. doi: 10.1128/aem.03006-05, PMID: 16820507 PMC1489311

[ref16] Edgar R. C. (2010). Search and clustering orders of magnitude faster than BLAST. Bioinformatics 26, 2460–2461. doi: 10.1093/bioinformatics/btq461, PMID: 20709691

[ref17] Foy R. H. Gibson C. E. Smith R. V. (2007). The influence of daylength, light intensity and temperature on the growth rates of planktonic blue-green algae. Br. Phycol. J 11, 151–163. doi: 10.1080/00071617600650181

[ref18] Fuhrman J. A. Steele J. A. Hewson I. Schwalbach M. S. Brown M. V. Green J. L.. (2008). A latitudinal diversity gradient in planktonic marine bacteria. Proc. Natl. Acad. Sci. U. S. A. 105, 7774–7778. doi: 10.1073/pnas.0803070105, PMID: 18509059 PMC2409396

[ref19] Gan L. Zheng J. Xu W.-H. Lin J. Liu J. Zhang Y.. (2022). Deciphering the virulent *Vibrio harveyi* causing spoilage in muscle of aquatic crustacean *Litopenaeus vannamei*. Sci. Rep. 12:16296. doi: 10.1038/s41598-022-20565-1, PMID: 36175476 PMC9522882

[ref20] Gelsomino A. Keijzer-Wolters A. C. Cacco G. Van Elsas J. D. (1999). Assessment of bacterial community structure in soil by polymerase chain reaction and denaturing gradient gel electrophoresis. J. Microbiol. Methods 38, 1–15. doi: 10.1016/s0167-7012(99)00054-8 10520580

[ref21] Gill S. R. Pop M. Deboy R. T. Eckburg P. B. Turnbaugh P. J. Samuel B. S.. (2006). Metagenomic analysis of the human distal gut microbiome. Science 312, 1355–1359. doi: 10.1126/science.1124234, PMID: 16741115 PMC3027896

[ref22] Gray S. B. Classen A. T. Kardol P. Yermakov Z. Mille R. M. (2011). Multiple climate change factors interact to Alter soil microbial community structure in an old-field ecosystem. Soil Sci. Soc. Am. J. 75, 2217–2226. doi: 10.2136/sssaj2011.0135

[ref23] Hernandez-Perez A. Noonin C. Soderhall K. Soderhall I. (2020). Environmental concentrations of sulfamethoxazole increase crayfish *Pacifastacus leniusculus* susceptibility to white spot syndrome virus. Fish Shellfish Immunol. 102, 177–184. doi: 10.1016/j.fsi.2020.04.022, PMID: 32311459

[ref24] Hooper D. U. Adair E. C. Cardinale B. J. Byrnes J. E. K. Hungate B. A. Matulich K. L.. (2012). A global synthesis reveals biodiversity loss as a major driver of ecosystem change. Nature 486:105. doi: 10.1038/nature11118, PMID: 22678289

[ref25] Hooper D. U. Chapin F. S. Ewel J. J. Hector A. Inchausti P. Lavorel S.. (2005). Effects of biodiversity on ecosystem functioning: a consensus of current knowledge. Ecol. Monogr. 75, 3–35. doi: 10.1016/j.drugalcdep.2010.01.006

[ref26] Hou M. Xiong J. Kai W. Ye X. Ran Y. Wang Q.. (2014). Communities of sediment ammonia-oxidizing bacteria along a coastal pollution gradient in the East China Sea. Mar. Pollut. Bull. 86, 147–153. doi: 10.1016/j.marpolbul.2014.07.031, PMID: 25110045

[ref27] Huang Z. Li X. Wang L. Shao Z. (2014). Changes in the intestinal bacterial community during the growth of white shrimp *Litopenaeus vannamei*. Aquacult. Res. doi: 10.1111/are.12628

[ref28] Iii F. Mcfarland J. Mcguire A. D. Euskirchen E. S. Ruess R. W. Kielland K. (2009). The changing global carbon cycle: linking plant–soil carbon dynamics to global consequences. J. Ecol. 97, 840–850. doi: 10.1111/j.1365-2745.2009.01529.x

[ref29] Iqbal M. Van Es H. M. Anwar Ul H. Schindelbeck R. R. Moebius-Clune B. N. (2014). Soil health indicators as affected by Long-term application of farm manure and cropping patterns under semi-arid climates. Int. J. Agric. Biol. 65, 206–250. doi: 10.1071/CP13342

[ref30] Li X. Liu L. Zhu Y. Zhu T. Wu X. Yang D. (2021). Microbial community structure and its driving environmental factors in black carp (*Mylopharyngodon piceus*) aquaculture pond. Water 13:3089. doi: 10.3390/w13213089

[ref31] Lin G. Sun F. Wang C. Li Z. Zhang X. (2017). Assessment of the effect of *Enteromorpha prolifera* on bacterial community structures in aquaculture environment. PLoS One 12. doi: 10.1371/journal.pone.017979210.1371/journal.pone.0179792.g00110 PMC5526538 28742878

[ref32] Liu Q. Long Y. A. Li B. Zhao L. L. Luo J. Xu L.. (2020). Rice-shrimp culture: a better intestinal microbiota, immune enzymatic activities, and muscle relish of crayfish (*Procambarus clarkii*) in Sichuan Province. Appl. Microbiol. Biotechnol. 104, 9413–9420. doi: 10.1007/s00253-020-10797-4, PMID: 32949278

[ref33] Liu Z. Mx L. Xl K. Wang M. Df Z. (2018). Correlation between microflora structure in intestinal tract and aquaculture environment of tilapia (*Oreochromis niloticus*) and streptococcicosis. J. Fish. China 42, 1635–1647. doi: 10.11964/jfc.20170910951

[ref34] Magoc T. Salzberg S. L. (2011). FLASH: fast length adjustment of short reads to improve genome assemblies. Bioinformatics 27, 2957–2963. doi: 10.1093/bioinformatics/btr507, PMID: 21903629 PMC3198573

[ref35] Moriarty D. (1997). The role of microorganisms in aquaculture ponds. Aquaculture 151, 333–349. doi: 10.1016/S0044-8486(96)01487-1

[ref36] Musthaq S. K. S. Kwang J. (2014). Evolution of specific immunity in shrimp – a vaccination perspective against white spot syndrome virus. Dev. Comp. Immunol. 46, 279–290. doi: 10.1016/j.dci.2014.04.013, PMID: 24780624

[ref37] Nayak S. K. (2010). Role of gastrointestinal microbiota in fish. Aquac. Res. 41, 1553–1573. doi: 10.1111/j.1365-2109.2010.02546.x

[ref38] Orwin K. H. Wardle D. A. Setl G. H. (2006). Context-dependent changes in the resistance and resilience of soil microbes to an experimental distrubance for three primary plant chronosequences. Oikos 112, 196–208. doi: 10.2307/3548572

[ref39] Panizzon J. P. Mussoi Macedo V. R. Machado V. Fiuza L. M. (2013). Microbiological and physical-chemical water quality of the rice fields in Sinos River's basin, southern Brazil. Environ. Monit. Assess. 185, 2767–2775. doi: 10.1007/s10661-012-2747-1, PMID: 22752964

[ref40] Rawls J. F. Mahowald M. A. Ley R. E. Gordon J. I. (2006). Reciprocal gut microbiota transplants from zebrafish and mice to germ-free recipients reveal host habitat selection. Cells 127, 423–433. doi: 10.1016/j.cell.2006.08.043, PMID: 17055441 PMC4839475

[ref41] Reynolds C. S. (2006). The ecology of phytoplankton: growth and replication of phytoplankton. Ecology 5, 178–238. doi: 10.1017/CBO9780511542145.00616634309

[ref42] Romo S. Fernández F. Ouahid Y. Barón-Sola N. (2012). Assessment of microcystins in lake water and fish (Mugilidae, Liza sp.) in the largest Spanish coastal lake. Environ. Monit. Assess. 184, 939–949. doi: 10.1007/s10661-011-2011-0 21472388

[ref43] Rungrassamee W. Klanchui A. Chaiyapechara S. Maibunkaew S. Tangphatsornruang S. Jiravanichpaisal P.. (2013). Bacterial population in intestines of the black Tiger shrimp (*Penaeus monodon*) under different growth stages. PLoS One 8:e60802. doi: 10.1371/journal.pone.0060802, PMID: 23577162 PMC3618293

[ref44] Rungrassamee W. Klanchui A. Maibunkaew S. Chaiyapechara S. Karoonuthaisiri N. (2014). Characterization of intestinal Bacteria in wild and domesticated adult black Tiger shrimp (*Penaeus monodon*). PLoS One 9:e91853. doi: 10.1371/journal.pone.0091853, PMID: 24618668 PMC3950284

[ref45] Si G. Peng C. Yuan J. Xu X. Zhao S. Xu D.. (2017). Changes in soil microbial community composition and organic carbon fractions in an integrated rice-crayfish farming system in subtropical China. Sci. Rep. 7:2856. doi: 10.1038/s41598-017-02984-7, PMID: 28588212 PMC5460161

[ref46] Silva K. Salles J. F. Seldin L. Elsas J. (2003). Application of a novel Paenibacillus-specific PCR-DGGE method and sequence analysis to assess the diversity of Paenibacillus spp. in the maize rhizosphere. J. Microbiol. Methods 54, 213–231. doi: 10.1016/S0167-7012(03)00039-3, PMID: 12782377

[ref47] Singh S. R. Yadav P. Singh D. Tripathi M. K. Bahadur L. Singh S. P.. (2020). Cropping systems influence microbial diversity, soil quality and crop yields in indo-Gangetic plains of India. Eur. J. Agron. 121:126152. doi: 10.1016/j.eja.2020.126152

[ref48] Srivastava J. K. Chandra H. Kalra S. Mishra P. Yadav P. (2016). Plant–microbe interaction in aquatic system and their role in the management of water quality: a review. Appl. Water Sci. 7, 1079–1090. doi: 10.1007/s13201-016-0415-2

[ref49] Sun Z. Guo Y. Li C. Cao C. Yuan P. Zou F.. (2019). Effects of straw returning and feeding on greenhouse gas emissions from integrated rice-crayfish farming in Jianghan plain, China. Environ. Sci. Pollut. Res. 26, 11710–11718. doi: 10.1007/s11356-019-04572-w, PMID: 30806926

[ref50] Sun Y. Han W. Liu J. Huang X. Zhou W. Zhang J.. (2020). Bacterial community compositions of crab intestine, surrounding water, and sediment in two different feeding modes of *Eriocheir sinensis*. Aquacult. Rep. 16:100236. doi: 10.1016/j.aqrep.2019.100236

[ref51] Sun F. Wang Y. Wang C. Zhang L. Zheng Z. (2019). Insights into the intestinal microbiota of several aquatic organisms and association with the surrounding environment. Aquaculture 507, 196–202. doi: 10.1016/j.aquaculture.2019.04.026

[ref52] The People's Republic of China Ministry of Agriculture. (2022). China fishery statistical yearbook 2022. Beijing: China Agriculture Press.

[ref53] Tilman R. Reich P. B. Knops J. Wedin D. Mielke T. Lehman C. (2001). Diversity and productivity in a long-term grassland experiment. Science 294, 843–845. doi: 10.1126/science.1060391, PMID: 11679667

[ref54] Van Rooyen J. M. Abratt V. R. Belrhali H. Sewell T. (2011). Crystal structure of type III glutamine Synthetase: surprising reversal of the inter-ring Interface. Structure 19, 471–483. doi: 10.1016/j.str.2011.02.001, PMID: 21481771

[ref55] Vasconcelos V. M. Pereira E. (2001). Cyanobacteria diversity and toxicity in a wastewater treatment plant (Portugal). Water Res. 35, 1354–1357. doi: 10.1016/S0043-1354(00)00512-1, PMID: 11268858

[ref56] Vazquez L. Alpuche J. Maldonado G. Agundis C. Pereyra-Morales A. Zenteno E. (2009). Immunity mechanisms in crustaceans. Innate Immun. 15, 179–188. doi: 10.1177/1753425909102876 19474211

[ref57] Xie J. Hu L. Tang J. Wu X. Chen X. (2011a). Ecological mechanisms underlying the sustainability of the agricultural heritage rice–fish coculture system. Proc. Natl. Acad. Sci. U. S. A. 108, E1381–E1387. doi: 10.1073/pnas.1111043108, PMID: 22084110 PMC3250190

[ref58] Xie J. Hu L. Tang J. Wu X. Xin C. (2011b). Ecological mechanisms underlying the sustainability of the agricultural heritage rice–fish coculture system. Proc. Natl. Acad. Sci. U. S. A. 108, 19851–19852. doi: 10.1073/pnas.1111043108 PMC3250190 22084110

[ref59] Xiong J. Zhu J. Zhang D. (2014). The application of bacterial indicator phylotypes to predict shrimp health status. Appl. Microbiol. Biotechnol. 98, 8291–8299. doi: 10.1007/s00253-014-5941-y, PMID: 25042597

[ref60] Xiujuan Y. Xiangju H. Ziqiao D. Linkun Y. (2022). Crayfish industry report 2022. China Fisheries 10:127. doi: 10.28152/n.cnki.ncyeb.2022.000127

[ref61] Yang C. Shuang C. Jin-Yu T. Yu T. Qiou-Yuan L. Zhi-Peng X.. (2021). Characteristics and differences of rice yield, quality, and economic benefits under different modes of comprehensive planting-breeding in paddy fields. Acta Agron. Sin. 47, 1953–1965. doi: 10.3724/sp.J.1006.2021.02068

[ref62] Zeng Y. (2020). *Aeromonas hydrophila*, one reason causing the death of freshwater crayfish *Procambarus clarkii* (Girard, 1852). Iran. J. Fish. Sci. 19, 1770–1779. doi: 10.22092/ijfs.2019.118282

[ref63] Zhang D. Wang X. Xiong J. Zhu J. (2014). Bacterioplankton assemblages as biological indicators of shrimp health status. Ecol. Indic. 38, 218–224. doi: 10.1016/j.ecolind.2013.11.002

[ref64] Zhang X. Xu M. Shi F. (2020). Impact of typical agricultural land use on the characteristics of soil microbial communities in the Nyingchi region of southeastern Tibet. J. Agro Environ. Sci. 39, 331–342.

